# Supplementary material for: Non-canonical role for Lpar1-EGFP subplate neurons in early postnatal mouse somatosensory cortex
Source: eLife. 2021 Jul 12;10:e60810. doi: 10.7554/eLife.60810 (PMC8294844; doi:10.7554/eLife.60810)
Supplement: Transparent reporting form [file elife-60810-transrepform.docx]

**Non-canonical role for *Lpar1-EGFP* subplate neurons in early postnatal
mouse somatosensory cortex**

Filippo Ghezzi^1,$^, Andre Marques-Smith^1,2,$^, Paul Anastasiades^1,3,#^, Daniel Lyngholm^1,#^, Cristiana Vagnoni^1,#^, Alexandra Rowett^1^, Gokul Parameswaran^1^, Anna Hoerder-Suabedissen^1^, Yasushi Nakagawa^4^, Zoltán Molnár^1^, Simon Butt^1,$,^*

1. Department of Physiology, Anatomy and Genetics, Sherrington Building, University of Oxford, South Parks Road, Oxford, OX1 3PT, UK.

2. Current address: CoMind Technologies, 5-7 Mandeville Place, London, W1U 3AY, UK

3. Current address: Department of Translational Health Sciences, University of Bristol, Dorothy Hodgkin Building, Whitson Street, Bristol, BS1 3NY, UK

4. Department of Neuroscience, University of Minnesota, Minneapolis, MN 55455, USA

* Corresponding author

^$^ These authors contributed equally to the manuscript

\# These authors contributed equally to the manuscript

**ABSTRACT [150]**

Subplate neurons (SPNs) are thought to play a role in nascent sensory processing in neocortex. To better understand how heterogeneity within this population relates to emergent function, we investigated the synaptic connectivity of *Lpar1-EGFP* SPNs through the first postnatal week in whisker somatosensory cortex (S1BF). These SPNs comprise of two morphological subtypes: fusiform SPNs with local axons, and pyramidal SPNs with axons that extend through the marginal zone. The former receive translaminar synaptic input up until the emergence of the whisker barrels; a timepoint coincident with significant cell death. In contrast, pyramidal SPNs receive local input from the subplate at early ages but then – during the later time window, acquire input from overlying cortex. Combined electrical and optogenetic activation of thalamic afferents identified that *Lpar1-EGFP* SPNs receive sparse thalamic innervation. These data reveal components of the postnatal network that interpret sparse thalamic input to direct the emergent columnar structure of S1BF.

**INTRODUCTION**

The emergence of function in the developing mammalian cerebral cortex is dependent on a diverse range of genetic and physiological processes that sculpt emergent network architecture. Fundamental research in animal models has revealed that transient neuronal circuits, observed in a restricted time window during early postnatal development, are a common feature of many cortical areas ^1, 2^. One of the first such transient circuits to be identified was that between subplate neurons (SPNs) and thalamo-recipient spiny stellate cells in layer L(4); a circuit demonstrated to play a role in the maturation of thalamocortical synapses ^1, 3, 4^. The subplate is a transient layer in the developing neocortex located between the emergent cortical plate and the underlying white matter ^5, 6^. It contains a diverse population of neuronal subtypes that differ in term of molecular markers ^7^, morphology ^8^, neurotransmitter identity^9^, and connectivity^10^. Electrophysiological studies performed in primary sensory areas suggest that SPNs are relatively mature when compared to cortical neurons in the more superficial cortical plate early in development ^8, 11^. As such they are regarded as key mediators of early spontaneous and sensory-evoked activity^4^, and direct circuit maturation^1^. While a large proportion of SPNs undergo programmed cell death during the first postnatal week in the mouse cortex^7^, the surviving SPNs form a thin, compact structure below layer 6, termed layer (L)6b in mature neocortex^12, 13^.

The canonical role of SPNs is to support the establishment of thalamocortical synapses onto L4 neurons^1, 3^. In support of this model, previous studies have reported that SPNs receive thalamocortical input prior to innervation of L4 neurons ^3, 14, 15, 16, 17^. In turn, SPNs are proposed to form feed-forward connections onto thalamo-recipient L4 neurons in a transient circuit that is eliminated upon establishment of the mature thalamocortical connectivity in L4 ^10, 18^. It was proposed that, by relaying thalamic inputs to L4 through SPNs, this connectivity pattern supports developmental plasticity mechanisms ^19^ prior to the appearance of the definitive cortical architecture in primary sensory areas e.g. ocular dominance in primary visual cortex (V1) ^3^, barrel field formation in primary somatosensory cortex (S1BF) ^4^, and tonotopic organization in the primary auditory cortex (A1) ^20^. In parallel, SPNs also promote the maturation of cortical GABAergic neurons ^19^, pioneer cortico-thalamic projections ^21^, secrete proteins that control extracellular matrix composition, attract and guide thalamocortical fibres, regulate plasticity and myelination ^22^, and control the radial migration of cortical neurons at embryonic ages ^23^.

However a number of unresolved questions remain regarding SPN function in neonatal cortex: first, it remains unclear how physiological, morphological and molecular heterogeneity of subplate neurons contributes to these various roles. While previous studies performed in A1 have identified two distinct physiological populations of SPN: those that receive feedback glutamatergic input from L4 and a second cohort that only receives local input ^10^, this has not been explicitly linked to SPN identity *per se*. To this end, we have focused on a specific, genetically-defined SPN population - labelled by the *Lpar1-EGFP* transgene^7^ to understand to what extent this population represents a homogeneous subtype of SPN and better resolve the role of these cells in neonatal somatosensory cortex. Moreover, recent evidence suggests that while thalamic input is a determinant of columnar organisation in late embryonic somatosensory cortex^24^, such activity pre-dates the transition to the mature cytoarchitecture and columnar signalling unit^25^. We sought to understand the role that *Lpar1-EGFP* SPN circuits have in interpreting such information through the first week of postnatal life up until the end of the layer 4 critical period for plasticity (CPP) at around postnatal day (P)8 in the barrel field of mouse primary somatosensory cortex (S1BF). We demonstrated that *Lpar1-EGFP* SPNs represent two distinct subtypes: (1) transient (<P5) fusiform SPNs that receive columnar input from the more superficial cortical plate but whose axons and therefore output are restricted to the SP syncytium; (2) pyramidal SPNs that are found throughout the time period recorded (≤P8), that only receive local input from the SP network prior to P5 but whose axons traverse the full extent of the cortical plate to ramifying extensively through the marginal zone. Finally, we identify that thalamic input onto *Lpar1-EGFP* SPNs in S1BF is sparse throughout early postnatal life. We propose that fusiform *Lpar1-EGFP* SPNs are ideally placed to interpret and amplify sparse thalamic input alongside emergent signalling from the cortical plate, thereby providing a template - through their innervation of other SPNs including the *Lpar1-EGFP* pyramidal subtype - for the columnar circuit assembly up until the emergence of whisker barrels in L4 at ~P4-P5. Our data suggest that *Lpar1-EGFP* SPNs do not adhere to the canonical role reported for SPNs in primary sensory cortex, and supports the idea that SPNs have a variety of ways of assisting cortical circuit construction.

**RESULTS**

**Intrinsic electrophysiological and morphological diversity of *Lpar1-EGFP* subplate neurons**

*Lpar1-EGFP* SPNs form a layer of 2-3 cells deep adjacent to the white matter tract in neonatal S1BF (**Figure 1a**). As a first pass to understanding the contribution of these neurons to neonatal circuits of S1BF we recorded the intrinsic electrophysiological profiles of 103 SPNs from postnatal day (P)1 to P8. We established that SPNs had an intrinsic electrophysiological profile consistent with regular firing pyramidal cells (**Figure 1b,c**) by injecting both depolarizing and hyperpolarizing current steps (500ms) of increasing amplitude into cells recorded at resting membrane potential in current clamp configuration. Analysis of passive (**Figure 1d-f**) and active (**Figure 1g-i**) properties revealed a progressive maturation of intrinsic properties across the ages tested broadly in line with previous reports ^18^. Specifically, we observed a statistically significant decrease in input resistance (RI_N_) (**Figure 1e)** and membrane time constant (**Figure 1f**), as well as increase in rheobase (**Figure 1g**), spike amplitude (**Figure 1h**), and maximum firing frequency (**Figure 1i**) over development. With a number of properties – membrane time constant (tau; **Figure 1f**), rheobase (pA; **Figure 1g**) and maximum firing frequency (Hz; **Figure 1i**), there was increased variability (±SD) with age (typically P5 onward) that suggests that not all SPNs mature at the same rate as development progressed.

Inclusion of Biocytin in the intracellular solution allowed us to reveal the morphologies of recorded SPNs (**Figure 2a**); both those assessed for intrinsic electrophysiological profile and subsequent optical stimulation experiments. In total we recovered 58 morphologies of 103 recorded SPNs that showed complete or near complete preservation of both axonal and dendritic arbor. It was evident from our reconstruction of 19 of these cells that *Lpar1-EGFP* SPNs fell into two categories based on dendritic arbor, and specifically the presence or absence of an apical dendrite (**Figure 2a,b**): (1) pyramidal-like SPNs with a prominent apical dendrite projecting into layer (L)6a; (2) fusiform SPNs that lacked an apical dendrite but instead exhibited bitufted dendrites that extended horizontally in the subplate and white matter tract. With the former, it was evident that the apical dendrite did not always extend perpendicular to the subplate as further revealed by analysis of the directionality of dendritic arbor (**Figure 2c**). Overlaying the axonal arbor of reconstructed morphologies revealed a further difference between these two populations: the axon of pyramidal SPNs (**Figure 2d**) ascended through the overlying cortex, with majority of cells projecting to the marginal zone/L1 (n = 9/12 cells, with the remaining 3 axons severed in L2). All of these cells had axonal collaterals – sometimes extensive – projecting within the subplate (SP/L6b) or adjacent L6a and the (**Figure 2d**). In contrast the axon of fusiform cells was largely restricted to the SP with a few collaterals extending into L6a (**Figure 2e**) and no axon projecting to more superficial layers (n = 7/7 cells). Both cell types had extensive, but relatively simple, axonal arbors that often extended beyond the field of the low power photomicrograph either through L1 (pyramidal) or SP (fusiform). It was evident that these long-range projections extended beyond S1BF to adjacent cortical areas such as secondary somatosensory cortex (S2). Morphologies were recovered across all recorded ages, however, the proportion of fusiform cells decreased from P5 onward (**Figure 2f**). Previous analysis of the neurotransmitter phenotype of *Lpar1-EGFP* SPNs was conducted at P7 ^7^, a time point when fusiform SPNs are no longer present in our sample (**Figure 2f**). To explore the possibility that these cells represent a transient GABAergic SP population^26^ we performed immunohistochemistry for GABA at P3 (**Figure 2g**). This confirmed that EGFP+ cells in the SP were all GABA-negative (0/79 Lpar1-EGFP SPNs co-expressed GABA) while the vast majority (84%; 16/19) of EGFP+ profiles in L5 were GABA+, consistent with our previous characterization of the SP^7^ and L5b interneuron populations^2^. These data identify *Lpar1-EGFP* SPNs as glutamatergic projection neurons that fall into two subtypes based on their morphology: fusiform cells that innervate the SP, and pyramidal cells whose axons extend through the entire depth of the developing cortex to ramify extensively through the margin zone/L1 (**Figure 2h**). We found no evidence of selectively targeted axonal innervation of L4 by either subtype at the ages recorded. However, we cannot discount innervation of L4 glutamatergic spiny stellate neurons via their apical dendrites extending to L1, which transiently exist prior to the end of the first postnatal week^27^. Finally, these data suggest that the fusiform population of *Lpar1-EGFP* SPN is a transient population of EGFP+ SPN not present in mature cortex

**Laser scanning photostimulation reveals dynamic synaptic integration of *Lpar1-EGFP* SPNs into local glutamatergic network**

We next used UV (355nm) laser photolysis of caged glutamate to map afferent synaptic input onto SPNs in acute *in vitro* cortical slices (**Figure 3a**). We mapped input using laser scanning photostimulation (LSPS) across the extent of a pseudo-random (50μm spaced) grid covering the depth of neocortex immediately above any given recorded cell. From the earliest time points recorded (P1-2) SPNs received distinct columnar glutamatergic input, either from SP and adjacent cortex (L6a) alone, or from both SP/L6a and more superficial cortex; patterns of innervation that we termed ‘local’ and ‘translaminar’ respectively (**Figure 3b**). The average laminar profile of local (n=15) and translaminar (n=8) SPNs at P1-2 revealed that the latter received input from the cortical plate (CP) absent in local SPNs (**Figure 3c**). This translaminar input became more prominent over the next two postnatal days (P3-4; **Figure 3d**) with the source primarily focused in the lower CP, presumptive L4. In contrast, at P5-6 we recorded relatively few translaminar neurons with the majority (84%) dominated by local SP/L6a input. The 3 cells defined as translaminar received afferent input from mostly infragranular pyramidal cells (**Figure 3e**). This trend continued in the cells recorded at P7-8 although the latter were diverse in input source such that the average laminar profile and map of translaminar cells resembled a diffuse columnar band of glutamatergic input (**Figure 3f**) across L4 and L5b.

While there were clear differences in laminar input profile, the average horizontal profile for both local and translaminar SPNs did not vary through development (**Figure 3g,h**). Recovered morphologies of SPNs mapped from P1-P4 (n=15) revealed that all the SPNs which received translaminar input were of the fusiform subtype (n=6). Two further fusiform and 7 pyramidal SPNs received local input. Of the 12 SPNs morphologies recovered from P5-8, 9 cells received local glutamatergic input. These were all of the pyramidal subtype with the exception of the only fusiform morphology recovered from our LSPS experiments during this later window. The remaining 3 pyramidal SPNs received translaminar input from L5. Taken together these data suggest that transient fusiform SPNs are the primary recipients of early translaminar input from the cortical plate (**Figure 3i**), up until P4 when they become less apparent in our sample. In parallel, pyramidal SPNs are dominated by local glutamatergic input from SP/L6a at early ages (**Figure 3i**), but acquire varied translaminar input from more superficial layers from P5 onward.

**Increased cell death in subplate and adjacent cortical layers at the P4 to P5 transition**

Subplate neurons are regarded as a transient neuronal population, but direct evidence in support of SPN programmed cell death is limited in murine models. One possible explanation is that rapid clearance of apoptotic neurons in the developing rodent brain might preclude histological identification of dying cells. Our morphological and LSPS data point to a possible 24-hour period from P4 to P5 during there is a change in the make-up of *Lpar1-EGFP* SPNs. To test if this is due to cell death, we performed immunohistochemistry at P3-4 and P5-6 to assess (1) the density of EGFP+ cells, (2) expression of the apoptotic marker cleaved Caspase-3 (Casp-3+), (3) pyknotic nuclei as evidenced by DAPI staining, (4) TUNEL staining to detect DNA breaks, in *Lpar1-EGFP* SPNs across these 2 time-windows (**Figure 4**). Caspase-3 staining was sparse at both time points but associated with pyknotic nuclei (**Figure 4a,b**). At P3-4 Casp-3+ cells were largely restricted to the white matter (**Figure 4a**), whereas at P5-6 while they were more widely distributed including in the SP. That said, we found only a couple of double positive EGFP+/Casp-3+ SPNs (n = 2 / 420 cells; 5 animals)( (**Figure 4b**). EGFP+ cells with pyknotic nuclei were however more evident, but the percentage EGFP+ cells with pyknotic nuclei did not differ between the two time points (**Figure 4c**), despite there being a significant decrease in the density of EGFP+ SPNs (**Figure 4d**). To further examine the latter observation, we performed a TUNEL stain as an alternative means of visualising apoptotic cells. This revealed a similar pattern of programmed cell death to our Casp-3 experiments, in that TUNEL+ cells were primarily located in the white matter at P3-4 (**Figure 4e**). However at P5-6, we observed a significant increase in TUNEL+ profiles (**Figure 4f**) that included *Lpar1-EGFP* SPNs (**Figure 4f,g**). Moreover, it was evident that this increase was not restricted to the subplate (**Figure 4f**) so we further quantified the density of TUNEL+ profiles across the white matter, subplate and adjacent infragranular layers at both time points; analysis which identified a surge in cell death at this later time point across the cortical layers sampled (**Figure 4h**). Overall, this suggests that while a small proportion of *Lpar1-EGFP* SPNs undergo cell death at the transition between P4 and P5, this is not specific to subplate and likely represent a wider reconfiguration of the circuit at the transition to columnar signalling in S1BF at this time^25^.

**Lpar1-EGFP SPNs receive distinct sources of GABAergic input including translaminar input from SST+ interneurons**

To understand if the observed changes around the P4 to P5 transition also involves early GABAergic circuits, we repeated our LSPS experimental strategy with the cell voltage clamped at the reversal potential for glutamate (E_Glut_)(**Figure 5a,b**). SPNs were pooled into two groups based on age: SPNs recorded prior to the P5 transition (P1-4; **Figure 5c-e**) and P5 onward (P5-8; **Figure 5f-h**). Similar to our previous assessment of glutamatergic input, it was evident that SPNs received either local (**Figure 5c,f**) or translaminar input (**Figure 5d,g**) across both time windows. Prior to P5, SPNs with local (**Figure 5c**) and translaminar (**Figure 5d**) input were evident in similar numbers with the latter receiving prominent columnar input from L5 (**Figure 5b,d,e**). Local GABAergic input was distributed through SP and adjacent L6a (**Figure 5c**). The average translaminar input (**Figure 5e**) revealed largely complementary distributions in GABAergic input for these two populations. From P5 onward we observed primarily local GABAergic synaptic input onto SPNs (n=11/16)(**Figure 5f**) with the source of translaminar GABAergic input highly variable in location resulting in a diffuse average input profile (**Figure 5g**) with a more-or-less even distribution across the depth of cortex (**Figure 5h**). Unlike glutamatergic input, the horizontal or columnar input was evenly spread for GABAergic input with the exception of translaminar input from P1-P4 (**Figure 5i,j**). Indeed translaminar GABAergic input onto SPNs prior to the emergence of whisker barrels at ~P5 was highly focused within the immediate column (**Figure 5i**). Recordings in the early time window yielded 7 morphologies of which 4 were fusiform cells that received translaminar input; the remaining 3 recovered neurons were the pyramidal subtype of which 2 received local input (**Figure 5k**). At the later age (P5-P8), we only recovered pyramidal SPN morphologies for both local and translaminar GABAergic input (n=4).

Somatostatin (SST+) interneurons form a key component of early postnatal translaminar circuits^2, 28^ and have been shown to drive synapse and circuit maturation^29, 30^. To test if these interneurons (INs) influence SPNs and the circuit transition observed around P5 we first conditionally expressed Channelrhodopsin2 (ChR2) in SST+ interneurons by crossing mice homozygous for the *Ai32* (ChR2) reporter allele onto our *Lpar1-EGFP* background that was also homozygous for the *SST-Cre* driver line to generate *Lpar1-EGFP;SSTCre;Ai32* offspring. We then used wide field blue light (470nm) illumination to evoke SST+ IN IPSCs in EGFP+ SPNs voltage clamped at E_Glut_ at P3-4 (n=7) and P5-6 (n=5). We observed an increase in IPSC amplitude across all laser powers tested greater or equal to minimal stimulation in the P5-6 when compared to the P3-4 time window (**Figure 6a**). To test if the increase in amplitude was a result of either increased quantal size or number of innervations we repeated these experiments in ACSF in which extracellular Ca^2+^ was replaced with strontium (Sr^2+^). Incubation with ACSF containing Sr^2+^ (Sr-ACSF) leads to asynchronous vesicular release at the presynaptic terminal providing a reasonable estimate of quantal size^31, 32^. In our hands incubation of neonatal SPNs in Sr-ACSF resulted in asynchronous release observed at minimal stimulation (**Figure 6b**) and a significant difference in ChR2-dependent IPSC amplitude between control and Sr-ACSF conditions (**Figure 6c**). However we observed no difference in the amplitude of IPSCs recorded in Sr-ACSF between P3-4 and P5-6 time windows (**Figure 6d**) despite the significant difference in amplitude under control conditions (**Figure 6b,c**). This suggests the observed increase in amplitude at this time point results from an increase in innervation by SST+ INs rather than an increase in quantal size.

Having established that SPNs received SST+ interneuron input through the first postnatal week, we next employed conditional expression of the P2x2 receptor – an optogenetic actuator that we have previously used in conjunction with uncaging of ATP^28^, that allows us to map the somatic location of presynaptic SST+ interneurons. We performed LSPS uncaging of ATP over the 50µm spaced pseudorandom grid to assess if the L5 translaminar input observed from P1-P4 originated from SST+ INs. Our analysis revealed two distinct input profiles for SPNs at this time (**Figure 6e,f**): local SP/L6a *versus* translaminar infragranular SST+ interneuron input. Similar to our previous findings with both glutamatergic and global GABAergic input, local input SPNs were pyramidal cells (3/3 recovered morphologies) whereas SPNs that received translaminar synaptic input were predominantly fusiform (2/3). The presence of GABAergic input onto SPNs from infragranular SST+ interneurons precedes our previously reported reciprocal connection between these SST+ cells and L4 spiny stellate neurons during the L4 critical period for plasticity (P4-P9)^2^ and then onto L2/3 pyramidal cells during the emergence of L4 to L2/3 feed-forward connections^28, 33^. Taken together, this evidence suggests that infragranular SST+ interneurons sequentially innervate thalamo-recipient layers through early postnatal life in S1BF.

**Sparse thalamocortical input onto early postnatal *Lpar1-EGFP* SPNs in S1BF**

SPNs are thought to play an important role in early thalamic integration in primary sensory cortices. To assess the role that *Lpar1-EGFP* SPNs play in the early sensory circuit we first used electrical stimulation of the ventrobasal complex (VB) of the thalamus while recording from EGFP+ SPNs in acute *in vitro* thalamocortical slice preparation (**Figure 7a**). Electrical stimulation evoked excitatory postsynaptic currents (EPSCs) in the majority (76%) of SPNs recorded across the time window studied, although there was a drop in incidence between the P1-4 and P5-8 (chi-square test X2 (1, N = 56) = 5.364, p = 0.021) time window (**Figure 7b**); an absence of thalamic input onto any given SPNs was only recorded if TC-EPSCs were observed in other SPNs or layer 4 neurons in the same thalamocortical slice. We observed no antidromic potentials in recorded EGFP+ SPNs. Analysis of the amplitude of the minimal electrical stimulation EPSC (**Figure 7c**) identified a significant change in variance between these times but no difference in amplitude (P = 0.08; two-tailed t-test, t=1.92, df=11.19). To further identify putative thalamocortical EPSCs (TC-EPSCs) we recorded the latency of the evoked EPSC, jitter (standard deviation in latency, ms), and amplitude for the minimal stimulation EPSC for 53 SPNs. All EPSCs with a latency >10ms and/or jitter >1.0ms (**Figure 7d**) were then excluded from our analysis leaving 32 SPNs that could be further divided into two populations based on 10-90% rise time and amplitude (**Figure 7e**): type 1 (n=9), large amplitude (55.3 pA ± SD 14.3), low jitter (0.20 ms ± SD 0.07), *versus* type 2 (n=23), small amplitude (14.9 mV ± SD 5.8), high jitter (0.41 ms ± SD 0.17) EPSCs (**Figure 7f**). Whether both populations represent TC-EPSCs^32, 34^ was unclear from our electrical stimulation of VB in part because both EPSC types conform to a previous criteria used to distinguish TC-EPSCs from antidromic cortico-thalamic EPSCs, namely that TC-EPSCs exhibit standard deviation in jitter <1.0ms ^35^. However thalamic connectivity could be as low as 17% if type 1 EPSCs recorded in SPNs represent true orthodromic TC-EPSCs; considerably lower than connectivity reported in previous studies of SP in early postnatal ages.

Given the disparity with previous reports we decided to employ optogenetics in parallel with electrical simulation of VB to unequivocally identify EPSCs arising from thalamic input (**Figure 7g**). We conditionally expressed ChR2 in thalamic nuclei using the *Olig3* Cre driver line that causes recombination throughout the thalamus early in development^36, 37^. To validate our optogenetic strategy at early postnatal ages, we first recorded from thalamic relay neurons in *Lpar1-EGFP;Olig3 Cre;Ai32* mice and established that we could evoke (1) reliable inward currents in whole cell patch clamp mode in response to blue, 470nm light (**Figure 7h**, top panel), and (2) time-locked action potentials in cell-attached mode (**Figure 7h**, bottom panel) from the earliest time points recorded (P1)(n=5 VB cells). We then recorded *Lpar1-EGFP* SPNs and tested for TC-EPSCs using both electrical and light stimulation protocols. Across the whole time window tested (P1-8) we obtained recordings under both stimulation protocols in 19 EGFP+ cells. Of these, 9 cells across all ages tested (P1-8) had short latency, low jitter (<1ms) EPSCs in response to electrical stimulation (type 1 and 2)(**Figure 7i)**. However only 2 cells (11% of *Lpar1-EGFP* SPNs), with properties consistent with type 1 EPSCs, exhibited responses to both 470nm light and electrical stimulation; no EGFP+ cells showed synaptic responses to light alone. To establish if sparse thalamocortical connectivity is a property of the *Lpar1-EGFP* subtypes alone, we then recorded from non-EGFP+ SPNs. Using our combined electrical and optogenetic stimulation strategy, we identified thalamic input onto only 3 out of 15 (27%) non-EGFP+ SPNs in the P1-P4 time window. We therefore believe that the majority of EPSCs observed after electrical stimulation, termed type 2 here, arise from antidromic activation of cortico-thalamic projection neurons. Taken together these data suggest that *Lpar1-EGFP* SPNs in S1BF receive both thalamic and cortico-thalamic input in the first postnatal week, but that the former is relatively sparse, contacting only a small subset of the total population.

**DISCUSSION**

We have recorded from a genetically identified population of subplate neuron (SPN) through the first postnatal week to establish the contribution of this cell type to early circuits of somatosensory whisker barrel cortex (S1BF). Our data reveal that the *Lpar1-EGFP* SPN population comprises of two distinct subtypes with different somatodendritic morphologies and with afferent input throughout the first 4 postnatal days (P1-4): first, pyramidal SPNs that receive primarily local input at this stage from the subplate (SP) network, but whose axons traverse across all the layers of cortex to project horizontally via layer 1 (**Figure 8a**). Second, fusiform SPNs that receive translaminar input from more superficial cortical layers but whose axons are largely confined to the immediate layer and therefore output to the SP network (**Figure 8b**). Fusiform SPNs receive glutamatergic input from the cortical plate, including putative layer 4, as well as GABAergic input from infragranular SST+ interneurons; two signalling centers that form reciprocal connections through the L4 critical period of plasticity in S1BF^2^. During the later period (P5-8), the onset of which coincides with an increase in cell death across cortical layers, fusiform cells are encountered in significantly reduced numbers – indeed are absent in the P7-8 window. The remaining pyramidal SPNs are evenly split between those that are still dominated by local inputs and those that acquire an array of diverse inputs from across cortical layers, located both in the immediate and adjacent cortical columns. This diversification of synaptic input fits with the rapid transition to columnar signalling previously reported at P5 ^25^, and suggests that this time point represents the switch from transient SP to layer 6b (L6b) ^13^ in S1BF.

Our study identifies that *Lpar1-EGFP* SPNs in S1BF have an additional novel function that does not conform to the canonical model for SPNs established across sensory cortices, wherein SPNs act as relay cells for thalamic input to L4 ^1, 4, 38^. We have tested thalamic engagement with *Lpar1-EGFP* SPNs using combined electrical and optogenetic stimulation of thalamic afferent fibres and found that the incidence of connectivity onto postnatal *Lpar1-EGFP* SPNs to be as low as 11% in thalamocortical slices that otherwise showed good preservation of connectivity. This is at odds with a number of previous studies reliant exclusively on electrical stimulation^18, 39^ which obtained levels of connectivity approaching our initial electrical stimulation paradigm. Recordings from non- *Lpar1-EGFP* subplate neurons suggest that this is not a property of this genetically defined population alone. As such, it is definitely worth revisiting perinatal thalamic engagement with the cortex using optogenetic approaches given that conditional expression of ChR2 in thalamic nuclei allows unequivocal discrimination of thalamocortical versus corticothalamic input without possible antidromic activation. Differences in the level of thalamic engagement aside, our data are consistent with the model that thalamic input is amplified via the subplate network^40^ and onward communicated via pyramidal SPNs to more superficial layers of cortex via layer 1. Indeed, our study builds on a number of studies that advanced our understanding of the role of subplate from a simple staging post for initial thalamic innervation to a critical mediator of plasticity and amplifier of thalamic input; a role that is fulfilled by two distinct morphological subtypes. However how this amplified signal is relayed to the overlying cortex is unclear beyond that it is likely mediated by the pyramidal subtype with axons ramifying in the marginal zone. None of our recovered morphologies suggest dense innervation of L4 *per s*e by this particular genetically-defined subpopulation of SPN^39^. Indeed this and our extensive mapping of S1BF using LSPS through early postnatal life^28, 41^ provide little evidence that there is a privileged route of connectivity between SP and L4 for a protracted period during early postnatal life in this primary sensory area, in contrast to other sensory modalities.

We targeted a genetically-defined population of SPN using the *Lpar1-EGFP* transgenic mouse line (GENSAT). Lpar1 (Edg2) is one of a number of markers previously shown to delineate SPN diversity^7^ with the cohort labelled by this line one of the earliest born subtypes with peak neurogenesis at embryonic day (E)11.5. EGFP expression is evident in this population at embryonic ages^7^, increasing to label both SP and non-SP neurons by the end of the first postnatal week^2^. This reported increase in strength of EGFP expression in SPNs through early postnatal life would seem to preclude the possibility that the fusiform SPN subtype down-regulate EGFP at P5. The most parsimonious explanation – that reconciles our observation of fusiform *Lpar1-EGFP* cells as a ‘classical’ transient SPN population with the fact that there is no significant decrease in the number of EGFP+ SPNs over this time window^7^ – is that this subtype represent only a small fraction of the EGFP+ SPN number. Our observed increase in the prevalence of the pyramidal subtype of EGFP+ SPNs over development has been documented by others^12^. Finally the *Lpar1-EGFP* transgenic is an excellent tool for targeting and recording these two morphological variants of SPN. However, our knowledge of the efferent targets of these cells is, in absence of a conditional genetic strategy, limited to a purely morphological assessment. Alternative genetic approaches such as CRE-DOG^42^ although useful to researchers targeting EGFP+ neuronal populations in the adult cortex^43^ do not provide a viable means of targeting the *Lpar1-EGFP* cells within the first postnatal week. What is evident from our analysis is that the two subtypes present in the earlier time window target completely different layers within the developing cortical plate, highly suggestive of different roles within the early circuitry of S1BF.

Laser scanning photostimulation (LSPS) has been used previously in conjunction with glutamate uncaging to probe the early SP circuits of primary auditory cortex (A1)^10, 15^. Similar to these reports, we find SPNs that receive local and translaminar input. However the temporal dynamics of the connections that we observe in S1BF, notably the early afferent input from L4, are quite different to those reported for A1 wherein L4 input emerges in the second postnatal week^10^. This disparity in timing could underpin differences in the role of SP in circuit maturation between sensory areas and suggests that the cytoarchitecture of sensory areas differs from the onset.

In recent years optical approaches have extended our knowledge of the early circuit of somatosensory cortex. It is evident that prenatal spontaneous thalamic activity plays an instrumental role in determining the columnar organisation of S1BF^24^. Shortly before birth thalamic afferent fibres are restricted to the subplate and thalamic stimulation elicits activity that spreads laterally through this and immediate adjacent infragranular layers^17^. It is possible that at these early ages thalamic innervation of SPNs is widespread in S1BF and that the role of such spontaneous activity is to competitively select the sparse SPNs that will maintain thalamic innervation into the first few postnatal days; a time point by which sensory activity has already transitioned to the overlying cortical layers in S1BF^24^. Our data suggest that *Lpar1-EGFP* pyramidal SPNs are likely conduits for such activity, recruiting pyramidal cells and GABAergic interneurons in more superficial cortex^14, 44^ via their L1 axon collaterals. In turn, neurons in the superficial cortical layers provide feedback columnar glutamatergic and GABAergic synaptic input onto transient fusiform SPNs thus completing the circuit (**Figure 8c**). This indirect mechanism could provide the necessary framework for the interpretation of early thalamic signals resulting in columnar organization. Indeed, such a mechanism coupled with the sparse nature of thalamic engagement with the subplate, represents a plausible substrate to ensure the emergence of spatially distinct columnar circuits in S1BF. Moreover, relay of thalamic input via L1 moves away from a L4-centric view of early thalamic engagement, more in line with distributed thalamocortical input across all 6 layers of neocortex^45^. Our findings provide insight into the earliest neuronal networks of somatosensory cortex, highlighting transient glutamatergic and GABAergic circuits that are essential for the emergence of normal perception.

**METHODS**

| **Key Resources Table** | | | | |
| --- | --- | --- | --- | --- |
| **Reagent type (species) or resource** | **Designation** | **Source or reference** | **Identifiers** | **Additional information** |
| Strain, strain background (*Mus musculus*, male) | *Tg(Lpar1-EGFP)GX193Gsat* | GENSAT Project at Rockefeller University | MGI:4847204; transgene insertion GX193 | Y Chromo- some linked |
| Strain, strain background (*M. musculus*, mixed sex) | *Sst^tm2.1(cre)Zjh^/J* | The Jackson Laboratory | Stock No: 013044;  RRID:IMSR_JAX:013044 | Maintained in Butt lab on C57BL/6 background |
| Strain, strain background (*M. musculus*, mixed sex) | *Olig3^tm1(cre)Ynka^* | Prof. Yasushi Nakagawa  University Minnesota |  |  |
| Strain, strain background (*M. musculus*, mixed sex) | *Gt(ROSA)26Sor^tm32(CAG-COP4*H134R/ EYFP)Hze^ /J* | The Jackson Laboratory | Stock No: 012569; RRID:IMSR_JAX:012569 | Maintained in Butt lab on C57BL/6 background |
| Strain, strain background (*M. musculus*, mixed sex) | *R26::P2x2r-EGFP* (floxed-stop-rat P2x2 receptor) | Prof. Gero Miesenböck (Oxford) |  | Maintained in Butt lab on C57BL/6 background |
| Antibody | Chicken anti-GFP  (polyclonal) | Abcam | ab13970  RRID: AB_300798 | (1:250) |
| Antibody | Rabbit anti-GABA (polyclonal) | Sigma-Aldrich | Cat# A2052, RRID: AB_477652 | (1:1000) |
| Antibody | Guinea-pig anti-GABA (polyclonal) | Abcam | ab17413  RRID: AB_443865 | (1:1500) |
| Antibody | Rabbit anti-Caspase 3 antibody, active (cleaved) form (polyclonal) | Merck Millipore | AB3623  RRID: AB_91556 | (1:200) |
| Antibody | Goat anti-Chicken, Alexa Fluor 488 (polyclonal) | Abcam | ab150169  RRID: AB_2636803 | (1:1000) |
| Antibody | Goat anti-Rabbit, Alexa Fluor 546 (polyclonal) | Thermo-Fisher | A11035  RRID: AB_143051 | (1:1000) |
| Commercial assay or kit | In Situ Cell Death Detection Kit, TMR red | Roche,  Sigma-Aldrich | SKU: 12156792910;  Lot #: 45197500 |  |
| Chemical compound, drug | NaCl;  KCl;  NaHCO_3_;  NaH_2_PO_4_;  MgCl_2_;  CaCl_2_;  K-gluconate;  Li-GTP;  Mg-ATP;  HEPES;  Gluconic acid;  EGTA;  SrCl_2_ | Sigma-Aldrich | S7653  P4504  S6014  S3139  M2670  22,350-6  G4500  G5884  A9187  H3375  G1951  E4378  204463 |  |
| Chemical compound, drug | MNI-caged glutamate | Tocris Bioscience UK | Cat. No.: 1490. Lot no.: 48A/206152; 50A/255070; 50A/220558; 50A225070; 51A/240237; 51A/243753; 52A/249897; 52A/251627 | 100µM |
| Chemical compound, drug | DMNPE-caged ATP | Life Technologies UK | A1049 | 100µM |
| Chemical compound, drug | Biocytin | Sigma- Aldrich | B4261;  Lot no. SLCB0219; SLBS5344 |  |
| Chemical compound, drug | Streptavidin, Alexa Fluor 568 conjugate | Thermo-Fisher | S11226  RRID: AB_2315774 | (1:500) |
| Software, algorithm | GraphPad Prism Software | GraphPad Software | RRID: CR_002798 |  |
| Software, algorithm | ImageJ/Fiji | Fiji | RRID: SCR_002285 |  |
| Software, algorithm | pClamp 10 | Molecular Devices | RRID: [SCR_011323](https://scicrunch.org/resolver/SCR_011323) |  |
| Software, algorithm | MATLAB | Mathworks | RRID: SCR_001622 |  |
| Other | DAPI stain | Thermo-Fisher | D1306  RRID: AB_2629482 | (1:1000) |

### **Animal husbandry and use**

Animal care and experimental procedures were approved by the University of Oxford local ethical review committee and conducted in accordance with UK Home Office personal and project (70/6767; 30/3052; P861F9BB75) licenses under the Animals (Scientific Procedures) 1986 Act. The following mouse lines were used: *Lpar1-EGFP* (Tg(Lpar1-EGFP)GX193Gsat), *SST-ires-Cre* (Sst^tm2.1(cre)Zjh^/J), *Olig3 Cre* (Olig3^tm1(cre)Ynka^), *Ai32* (*Gt(ROSA)26Sor^tm32(CAG-COP4*H134R/EYFP)Hze^/J*), *R26::P2x2R-EGFP* (floxed-stop-rat P2x2 receptor). All experiments were performed blind to the mouse genotype with the exception of *Lpar1-EGFP* transgene which is Y chromosome-linked^7^. The date of birth was designated postnatal day (P)0.

**Acute *in vitro* slice preparation**

Acute brain slices were prepared as previously described^2^. Male mice (postnatal day 1 to 8; P1-P8) were anaesthetised with 4% isoflurane in 100% O_2_ and decapitated; the cerebral cortex was quickly dissected in ice-cold, oxygenated (95% O_2_ / 5% CO_2_) artificial cerebrospinal fluid (ACSF) of the following composition (in mM): 125 NaCl, 2.5 KCl, 25 NaHCO_3_, 1.25 NaH_2_PO_4_, 1 MgCl_2_, 2 CaCl_2_, 20 glucose (300-310 mOsm; all chemicals were purchased from Sigma unless otherwise stated). Coronal and thamalocortical slices (350-400 µm) including the primary somatosensory barrel cortex (S1BF) were cut in ice-cold ACSF through a vibratome (Vibratome 3000 Plus; The Vibratome Company) and allowed to recover in ACSF at room temperature (RT) for at least 1 h prior to electrophysiological recordings. Coronal slices were obtained by cutting the brain at an angle perpendicular to S1BF; thalamocortical slices were obtained according to established procedures with the angle varied according to developmental age^2, 46^.

**Whole-cell patch-clamp electrophysiology**

Slices containing S1BF were selected for electrophysiology experiments if they showed good preservation of the radial structure, as assessed by the presence of layer (L)5 pyramidal neuron apical dendrites extending to supragranular layers. *Lpar1-EGFP* subplate neurons (SPNs) were readily distinguished from *Lpar1-EGFP* GABAergic interneurons based on their localization in a thin layer of cells located between the cortical L6 and the underlying white matter, identified as the subplate (SP). Layer 6b could be detected as a thin, compact cell layer that could be distinguished from Layer 6a and white matter. Cells were selected ~50 µm below the slice surface and targeted for patch-clamp recordings guided through infrared-differential interference contrast (IR-DIC) microscopy using a 40x water-immersion objective. Whole-cell patch clamp electrophysiological recordings were performed at RT using a Multiclamp 700B amplifier and Digidata 1440A digitizer (Molecular Devices, USA). Patch pipettes were obtained from borosilicate glass microelectrodes (6-9 MΩ; Harvard Apparatus, UK), pulled through a PC-10 puller (Narishige, Japan). Electrodes were filled with either a K-based (128 mM K-gluconate, 4 mM NaCl, 0.3 mM Li-GTP, 5 mM Mg-ATP, 0.1 mM CaCl_2_, 10 mM HEPES; pH 7.2 with KOH; 280-290 mOsm) or Cs-based intracellular solution (100 mM gluconic acid, 0.2 mM EGTA, 5 mM MgCl_2_ 40 mM HEPES, 2 mM Mg-ATP, 0.3 mM Li-GTP; pH 7.2 with CsOH; 280-290 mOsm). Biocytin (0.3%) was included in the intracellular solution to allow the morphological reconstruction of recorded neurons. To study excitatory postsynaptic currents (EPSCs), SPNs were held at a holding potential (V_h_) of -60 mV; inhibitory postsynaptic currents (IPSCs) were recorded by voltage-clamping the cell near the equilibrium potential for glutamate (E_Glut_). For mapping of IPSC input, E_Glut_ was found empirically by uncaging glutamate in the proximity of the recorded cell and tuning the V_h_ until little or no net laser-induced direct postsynaptic current was observed. For optogenetic experiment V_h_ was set to 0 mV (corrected for calculated liquid junction potential of ~13 mV for the Cs-based intracellular solution). All recordings were sampled at 20 kHz and low-pass filtered online at 0.5 kHz. Cell input and series resistance (R_in_ and R_s_) were monitored throughout the duration of the recording without applying compensation; recordings were discarded when R_s_ exceeded 20% of its initial value.

Cells patched with the K-based intracellular solution were initially held in current-clamp configuration to record their intrinsic electrophysiological profile prior to LSPS experiments. Intrinsic electrophysiological properties were assessed using both depolarizing and hyperpolarizing current steps (500ms) of increasing amplitude applied from resting membrane potential; step size was adjusted based on the input resistance of the cell.

**Laser-scanning photostimulation: methods and analysis**

Laser-scanning photostimulation (LSPS) was performed according to the method previously described^41, 47^. This optical technique allows to stimulate neurons into a small portion of cortical tissue (~50µm) while recording the postsynaptic current in the target neurons. Thus, the location of any presynaptic neurons showing functional connectivity to the recorded one can be inferred by the location of the optical stimulation. Prior to LSPS, slices were incubated for a minimum of 6 mins. with high-divalent cation (HDC) ACSF of similar composition to the normal ACSF but with increased concentration (4 mM) of MgCl_2_ and CaCl_2_ and supplemented with 100µM MNI-caged glutamate (Tocris Bioscience, UK) for glutamate uncaging experiments. We perform LSPS experiments in HDC ACSF solution to reduce polysynaptic transmission and the occurrence of spontaneous synaptic currents. Mapping of cell-type-selective inputs were performed with an optogenetic strategy previously described^28^. In brief, the P2x2 receptor was conditionally expressed into SST+ interneurons and selectively stimulated by laser uncaging of DMNPE-caged ATP (100µM, Life Technologies, UK). LSPS was performed using an ultraviolet (UV) laser (DSPL-355/30) and a galvanometer targeting system (UGA-42, Rapp Optoelectronic GmbH, Germany) focused through a 10x Olympus objective. The stimulation grid was organized into 17x9 target spots (~50µm spatial resolution). Long-duration (100 ms), low-power (<2 mW at sample plane) laser pulses were fired in a pseudo-random order at 1-2 Hz frequency. In order to cover the whole extent of the cortical column, 2-3 LSPS grids were sequentially employed and properly aligned and averaged offline during data analysis. For each individual LSPS grid, a minimum of 3 runs were obtained and averaged.

Electrophysiological current traces were analysed with Minianalysis 6.0 (Synaptosoft Inc.) to extrapolate amplitude and onset time of all IPSCs or EPSCs recorded. Direct responses to glutamate were identified by their short latency and slow onset kinetics (time to peak ~100ms) and excluded from subsequent analysis. Only EPSCs characterized by fast onset kinetics were considered for further analysis if they occurred within the putative detection window for monosynaptic events, determined according to previously published criteria^41^ . For each laser spot of the grid, all events whose onset fell within this detection window were summed and then averaged with values from different runs of the same experiment. Final heatmaps were built through a customized Matlab (Mathworks, US) script. In order to allow the reconstruction of the layer profile on the input map, a photomicrograph of the grids relative to the slice preparation was acquired and layer boundaries were manually determined. Normalized heatmaps were generated by dividing value in each spot by the sum of all pixels. Linear profiles (layer and columnar) were obtained by summing all values for each line in individual heatmaps. Average maps were obtained by aligning each individual map to the SP/Layer 6a boundary and averaging corresponding pixels.

***In vitro* optogenetics stimulation**

Optogenetics experiments were performed by conditionally expressing Channelrhodopsin 2 (ChR2, via the *Ai32* reporter allele) in SST+ interneurons (using *SST-ires-Cre*) or thalamic relay neurons (using *Olig3 Cre*). Wide-field light stimulation was delivered through a 40x objective to focus blue (470nm LED, CoolLED, UK) light onto the recorded cell. For each recorded SPN, two light stimulation duration pulses (1 and 10 ms) were employed at multiple LED power intensity to ensure that the minimal stimulation and full range of activation was captured irrespective of developmental age. For each LED pulse duration and intensity, five pulses were administered at a 20s interval and the evoked postsynaptic current (PSC) recorded.

Data analysis was performed through a customised Matlab script. Light-evoked PSCs were analysed if their onset was detected within 25 ms from the onset of the light stimulus; the relatively long latency was used to account for developmental effects that may affect ChR2 expression. For events within the mono-synaptic detection window, multiple PSC features were extracted such as amplitude, latency, 10-90% rise time, and decay time constant (τ). In particular, the latency of the PSC was calculated from the onset of LED stimulation; the decay τ was found by fitting a mono-exponential curve to the decay phase of the PSC; percentage of PSC occurrence was calculated throughout the five sweeps at each LED intensity.

In a subset of experiments, recordings of light-evoked IPSCs from SST+ interneurons were performed in a modified HDC ACSF containing 4 mM SrCl_2_ to replace CaCl_2_. Due to the slow onset of its effects, slices were bathed in Sr^2+^-containing HDC for a minimum of 20 min before light stimulation and data collection (Gil et al., 1999).

**Electrical stimulation of thalamic afferents**

Thalamocortical (TC) afferent input to SPNs was tested using a bipolar microelectrode (Harvard Apparatus, UK) placed either in the ventrobasal nucleus (VB) of the thalamus or the internal capsule (IC) and connected to a current isolator (DS3, Digitimer Ltd, UK). The strength of the electrical stimulation was varied to find the minimal stimulation value^48, 49^, corresponding to EPSC evoked on ~50% of trials. The interstimulus interval was set at either 30 or 60s depending on developmental age. TC-EPSCs were considered if calculated standard deviation (jitter) of the EPSC latency was <1ms at minimal stimulation.

**Morphological reconstruction of recorded cells**

Following electrophysiological assessment, slices containing biocytin-filled cells were fixed in 4% paraformaldehyde (PFA; diluted in phosphate-buffered saline, PBS) overnight at 4^o^C. Slices were then rinsed in PBS and incubated in 0.05% PBST containing Streptavidin-Alexa568 (1:500; Molecular Probes, US) for 48-72 h at 4^o^C. Slices were then washed 3x 10mins. in PBS and mounted on histology slides with Fluoromount (Sigma) mounting medium.

Slices were imaged through an Olympus FV1200 confocal microscope equipped with 10x or 20x dry objective. Z-stack images were acquired in order to maximize imaging of all neuronal processes containing biocytin to allow the offline morphological reconstruction. Image analysis was performed with Fiji-ImageJ software (NIH): confocal images of filled cells were selected for morphological reconstruction, performed using the Simple Neurite Tracer plugin. Dendrite directionality was calculated using the Directionality plugin implemented in Fiji-ImageJ onto reconstructed dendritic morphologies.

**Immunohistochemistry and TUNEL stain**

Mice were terminally anaesthetized with pentobarbital (90 mg/kg) and transcardially perfused with 4% PFA in PBS. Dissected brains were incubated in PFA for 2 h at 4^o^C and then cryoprotected in 20% sucrose for 24 h at 4^o^C. Brains were then embedded into O.C.T. (VWR), frozen on dry ice and stored at -80^o^C. Each brain was sectioned into 14 to 16μm thick slices and mounted on histology slides; slides were stored at -20^o^C. Slides selected for immunohistochemistry were air-dried overnight at RT and washed 3x 10mins. at RT in PBS. Slides were then permeabilized for 30 min in 0.5% PBST (0.5 % Triton X-100 in PBS) and incubated in blocking solution (PBST 0.1%, Normal Goat Serum 5%) for 1 hour at RT. Primary antibodies used were chicken anti-GFP (ab13970, Abcam, dilution 1:250), rabbit anti-GABA (A2052, Sigma, 1:1000), guinea-pig anti-GABA (ab17413, Abcam, 1:1500) and rabbit anti-Caspase-3 (AB3623, Merck Millipore, 1:200). Slides were incubated in primary antibody solution overnight at 4^o^C. Slides were then washed 3x10 min at RT in PBS and subsequently incubated in secondary antibody (Goat anti-Chicken IgG Alexa Fluor 488 conjugate, Goat anti-Rabbit IgG Alexa Fluor 568 conjugate; diluted in blocking solution 1:1000) for 2 hours at RT. Finally, the slides were washed in PBS, counterstained with DAPI (diluted 1:1000 in PBS) for 3 minutes at RT and mounted with Fluoromont (Sigma). For the TUNEL stain, slides were processed for GFP immunohistochemistry as above, then the TUNEL stain was applied as per the supplier (Roche, SKU: 12156792910) instructions. Slides were then counterstained with DAPI and mounted as above. All slides were imaged through an Olympus FV1200 confocal microscope.

**Statistical analysis**

All results are expressed as mean ± standard error of the mean; n indicates the number of cells recorded. Statistical analysis was performed with Prism (GraphPad, US). Normality and equal variance tests were run to direct the appropriate statistical test choice for comparison of parametric versus non-parametric datasets. Multiple groups were compared with a two-way ANOVA test; *post hoc* multiple comparisons were performed with the Holm-Sidak method. Two parametric groups were compared with Student’s t-test whereas two non-parametric groups were compared with the Mann-Whitney rank sum test. Finally, cumulative frequency distributions were compared with the two-sample Kolmogorov-Smirnov test. Statistical significance was evaluated at P ≤ 0.05; for the Kolmogorov-Smirnov test P ≤ 0.01 was considered statistically significant.

**Acknowledgements**

Research in the Butt lab that contributed to this work was funded by the Medical Research Council (MRC)(MR/K004387/1), Biotechnology and Biological Sciences Research Council

(BB/P003796/1), Human Frontiers Science Program Organisation (CDA0023/2008-C) and Brain and Behavior Research Foundation (Narsad; ref. 19079). Studentships awarded to FG and AM-S were funded by the Wellcome Trust; PGA was funded by an Imperial College London studentship; CV was funded by an MRC studentship. Funding for equipment came from the Wellcome Trust (089286/Z/09/Z) and OUP John Fell Fund (AV6721-C8000). Work in the Molnár laboratory related to early cortical circuit formation was funded by the MRC (G00900901, MR/N026039/1), Royal Society and Anatomical Society.

**References**

1.	Kanold PO, Luhmann HJ. The subplate and early cortical circuits. *Annual review of neuroscience* **33**, 23-48 (2010).

2.	Marques-Smith A*, et al.* A Transient Translaminar GABAergic Interneuron Circuit Connects Thalamocortical Recipient Layers in Neonatal Somatosensory Cortex. *Neuron* **89**, 536-549 (2016).

3.	Kanold PO, Kara P, Reid RC, Shatz CJ. Role of subplate neurons in functional maturation of visual cortical columns. *Science* **301**, 521-525 (2003).

4.	Tolner EA, Sheikh A, Yukin AY, Kaila K, Kanold PO. Subplate neurons promote spindle bursts and thalamocortical patterning in the neonatal rat somatosensory cortex. *The Journal of neuroscience : the official journal of the Society for Neuroscience* **32**, 692-702 (2012).

5.	Kostovic I, Rakic P. Developmental history of the transient subplate zone in the visual and somatosensory cortex of the macaque monkey and human brain. *The Journal of comparative neurology* **297**, 441-470 (1990).

6.	Hoerder-Suabedissen A, Molnar Z. Development, evolution and pathology of neocortical subplate neurons. *Nature reviews Neuroscience* **16**, 133-146 (2015).

7.	Hoerder-Suabedissen A, Molnar Z. Molecular diversity of early-born subplate neurons. *Cerebral cortex* **23**, 1473-1483 (2013).

8.	Marx M, Feldmeyer D. Morphology and physiology of excitatory neurons in layer 6b of the somatosensory rat barrel cortex. *Cerebral cortex* **23**, 2803-2817 (2013).

9.	Boon J, Clarke E, Kessaris N, Goffinet A, Molnar Z, Hoerder-Suabedissen A. Long-range projections from sparse populations of GABAergic neurons in murine subplate. *The Journal of comparative neurology* **527**, 1610-1620 (2019).

10.	Viswanathan S, Bandyopadhyay S, Kao JP, Kanold PO. Changing microcircuits in the subplate of the developing cortex. *The Journal of neuroscience : the official journal of the Society for Neuroscience* **32**, 1589-1601 (2012).

11.	Luhmann HJ, Reiprich RA, Hanganu I, Kilb W. Cellular physiology of the neonatal rat cerebral cortex: intrinsic membrane properties, sodium and calcium currents. *J Neurosci Res* **62**, 574-584 (2000).

12.	Marx M, Qi G, Hanganu-Opatz IL, Kilb W, Luhmann HJ, Feldmeyer D. Neocortical Layer 6B as a Remnant of the Subplate - A Morphological Comparison. *Cerebral cortex* **27**, 1011-1026 (2017).

13.	Zolnik TA*, et al.* Layer 6b Is Driven by Intracortical Long-Range Projection Neurons. *Cell reports* **30**, 3492-3505 e3495 (2020).

14.	Friauf E, Shatz CJ. Changing patterns of synaptic input to subplate and cortical plate during development of visual cortex. *Journal of neurophysiology* **66**, 2059-2071 (1991).

15.	Zhao C, Kao JP, Kanold PO. Functional excitatory microcircuits in neonatal cortex connect thalamus and layer 4. *The Journal of neuroscience : the official journal of the Society for Neuroscience* **29**, 15479-15488 (2009).

16.	Molnar Z, Kurotani T, Higashi S, Yamamoto N, Toyama K. Development of functional thalamocortical synapses studied with current source-density analysis in whole forebrain slices in the rat. *Brain Res Bull* **60**, 355-371 (2003).

17.	Higashi S, Molnar Z, Kurotani T, Toyama K. Prenatal development of neural excitation in rat thalamocortical projections studied by optical recording. *Neuroscience* **115**, 1231-1246 (2002).

18.	Hanganu IL, Kilb W, Luhmann HJ. Functional synaptic projections onto subplate neurons in neonatal rat somatosensory cortex. *The Journal of neuroscience : the official journal of the Society for Neuroscience* **22**, 7165-7176 (2002).

19.	Kanold PO, Shatz CJ. Subplate neurons regulate maturation of cortical inhibition and outcome of ocular dominance plasticity. *Neuron* **51**, 627-638 (2006).

20.	Wess JM, Isaiah A, Watkins PV, Kanold PO. Subplate neurons are the first cortical neurons to respond to sensory stimuli. *Proceedings of the National Academy of Sciences of the United States of America* **114**, 12602-12607 (2017).

21.	McConnell SK, Ghosh A, Shatz CJ. Subplate neurons pioneer the first axon pathway from the cerebral cortex. *Science* **245**, 978-982 (1989).

22.	Kondo S, Al-Hasani H, Hoerder-Suabedissen A, Wang WZ, Molnar Z. Secretory function in subplate neurons during cortical development. *Frontiers in neuroscience* **9**, 100 (2015).

23.	Ohtaka-Maruyama C*, et al.* Synaptic transmission from subplate neurons controls radial migration of neocortical neurons. *Science* **360**, 313-317 (2018).

24.	Anton-Bolanos N*, et al.* Prenatal activity from thalamic neurons governs the emergence of functional cortical maps in mice. *Science* **364**, 987-990 (2019).

25.	Dupont E, Hanganu IL, Kilb W, Hirsch S, Luhmann HJ. Rapid developmental switch in the mechanisms driving early cortical columnar networks. *Nature* **439**, 79-83 (2006).

26.	Qu GJ, Ma J, Yu YC, Fu Y. Postnatal development of GABAergic interneurons in the neocortical subplate of mice. *Neuroscience* **322**, 78-93 (2016).

27.	Callaway EM, Borrell V. Developmental sculpting of dendritic morphology of layer 4 neurons in visual cortex: influence of retinal input. *The Journal of neuroscience : the official journal of the Society for Neuroscience* **31**, 7456-7470 (2011).

28.	Anastasiades PG*, et al.* GABAergic interneurons form transient layer-specific circuits in early postnatal neocortex. *Nat Commun* **7**, 10584 (2016).

29.	Oh WC, Lutzu S, Castillo PE, Kwon HB. De novo synaptogenesis induced by GABA in the developing mouse cortex. *Science* **353**, 1037-1040 (2016).

30.	Tuncdemir SN*, et al.* Early Somatostatin Interneuron Connectivity Mediates the Maturation of Deep Layer Cortical Circuits. *Neuron* **89**, 521-535 (2016).

31.	Oliet SH, Malenka RC, Nicoll RA. Bidirectional control of quantal size by synaptic activity in the hippocampus. *Science* **271**, 1294-1297 (1996).

32.	Gil Z, Connors BW, Amitai Y. Efficacy of thalamocortical and intracortical synaptic connections: quanta, innervation, and reliability. *Neuron* **23**, 385-397 (1999).

33.	Bureau I, Shepherd GM, Svoboda K. Precise development of functional and anatomical columns in the neocortex. *Neuron* **42**, 789-801 (2004).

34.	Luz LL, Currie SP, Daw MI. Alterations in the properties of neonatal thalamocortical synapses with time in in vitro slices. *PLoS One* **12**, e0171897 (2017).

35.	Rose HJ, Metherate R. Auditory thalamocortical transmission is reliable and temporally precise. *Journal of neurophysiology* **94**, 2019-2030 (2005).

36.	Vue TY*, et al.* Characterization of progenitor domains in the developing mouse thalamus. *The Journal of comparative neurology* **505**, 73-91 (2007).

37.	Vue TY*, et al.* Sonic hedgehog signaling controls thalamic progenitor identity and nuclei specification in mice. *The Journal of neuroscience : the official journal of the Society for Neuroscience* **29**, 4484-4497 (2009).

38.	Allendoerfer KL, Shatz CJ. The subplate, a transient neocortical structure: its role in the development of connections between thalamus and cortex. *Annual review of neuroscience* **17**, 185-218 (1994).

39.	Friauf E, McConnell SK, Shatz CJ. Functional synaptic circuits in the subplate during fetal and early postnatal development of cat visual cortex. *The Journal of neuroscience : the official journal of the Society for Neuroscience* **10**, 2601-2613 (1990).

40.	Luhmann HJ, Kilb W, Hanganu-Opatz IL. Subplate cells: amplifiers of neuronal activity in the developing cerebral cortex. *Front Neuroanat* **3**, 19 (2009).

41.	Anastasiades PG, Butt SJ. A role for silent synapses in the development of the pathway from layer 2/3 to 5 pyramidal cells in the neocortex. *The Journal of neuroscience : the official journal of the Society for Neuroscience* **32**, 13085-13099 (2012).

42.	Tang JC*, et al.* Cell type-specific manipulation with GFP-dependent Cre recombinase. *Nature neuroscience* **18**, 1334-1341 (2015).

43.	Naka A*, et al.* Complementary networks of cortical somatostatin interneurons enforce layer specific control. *Elife* **8**, (2019).

44.	Che A*, et al.* Layer I Interneurons Sharpen Sensory Maps during Neonatal Development. *Neuron* **99**, 98-116 e117 (2018).

45.	Feldmeyer D*, et al.* Barrel cortex function. *Prog Neurobiol* **103**, 3-27 (2013).

46.	Agmon A, Connors BW. Thalamocortical responses of mouse somatosensory (barrel) cortex in vitro. *Neuroscience* **41**, 365-379 (1991).

47.	Anastasiades PG, Marques-Smith A, Butt SJB. Studies of cortical connectivity using optical circuit mapping methods. *The Journal of physiology* **596**, 145-162 (2018).

48.	Raastad M, Storm JF, Andersen P. Putative Single Quantum and Single Fibre Excitatory Postsynaptic Currents Show Similar Amplitude Range and Variability in Rat Hippocampal Slices. *The European journal of neuroscience* **4**, 113-117 (1992).

49.	Isaac JT, Crair MC, Nicoll RA, Malenka RC. Silent synapses during development of thalamocortical inputs. *Neuron* **18**, 269-280 (1997).

**FIGURE LEGENDS**

**Figure 1 Intrinsic electrophysiological properties of Lpar1-EGFP SPNs.**

(a) Streptavidin (568nm) labelling of record Lpar1-EGFP SPNs in mouse S1BF at P2. (b) Superimposed electrophysiology traces recorded from one of the cells shown in panel (a) in response to hyperpolarizing and depolarizing threshold current injection. (c) Maximum firing frequency for the same cell; scale bar is the same for panels (b) and (c). (d-i) Summary data for 103 cells grouped according to age: P1-2 (n=22); P3-4 (n=27); P5-6 (n=24); P7-8 (n=30). The range of passive membrane properties recorded included (d) resting membrane potential (RMP)(mV); (e) Input resistance (RIN)(GΩ); (f) Membrane time constant (tau)(ms). Active properties included (g) current injection required for threshold spike (rheobase)(mV); (h) spike amplitude (mV); (i) maximum firing frequency (Hz). We observed a statistically significant difference between age groups for R_IN_ (ANOVA, F=21.17, P<0.001), decay time constant (Kruskal-Wallis test, KW=37.75, P < 0.001)**,** rheobase (Kruskal-Wallis test, KW=15.74, P < 0.01), spike amplitude (ANOVA, F=22.05, P<0.001), and maximum firing frequency (Kruskal-Wallis test, KW=43.15, P < 0.001) whereas no statistical significant difference was observed for resting membrane potential (ANOVA, F=1.74, P>0.05). Significant multiple comparisons of note are indicted in the relevant panels: *, 0.01<P<0.05; **, P<0.01.

**Figure 2 Two distinct morphologies of Lpar1-EGFP SPNs in S1BF.**

(a) Streptavidin labelled morphologies of 2 EGFP+ SPNs recorded at P3; arrowhead, pyramidal subtypes with apical dendrite projecting at ~45o into L6a; arrow, fusiform morphology with horizontal, bitufted dendrites largely restricted to the SP (Scale bar = 25μm). (b) Reconstructed (ImageJ) dendritic arbors of the cells shown (a) were used to calculate directionality with 90o indicative of vertically orientated dendrites and ~0o primarily horizontal dendrites (Scale bar = 25μm). (c) A difference was observed in the dendritic orientation of fusiform and pyramidal SPNs (Mann-Whitney U=5, fusiform n=7, median = 4.38; pyramidal n=12, median = 54.89, **P<0.001 two-tailed). Overlay of axonal arbors of (d) pyramidal (recovered between P1-P8) and (e) fusiform (recovered P1-P5) cells aligned on soma location (horizontal position indicated by the blue circle); approximate barrel location indicated by grey shaded areas for P5+ cells; scale bar 180μm. (f) Proportion of fusiform (grey) and pyramidal (black) Lpar1-EGFP SPNs over the first postnatal week. (g) Immunohistochemistry for EGFP (left panel) and GABA (middle panel); EGFP+/GABA+ cells indicated by the white arrows; right panel, overlay of EGFP (green) and GABA (red). (h) Schematic of the two morphological subtypes of Lpar1-EGFP SPN: pyramidal (Pyr) and fusiform SPNs encountered prior to P5; a putative synaptic connection between the two subtypes is indicated by the question mark. MZ, marginal zone; CP, cortical plate; SP, suplate.

**Figure 3 Synaptic integration of *Lpar1-EGFP* SPNs into the local cortical glutamatergic network.**

(a) LSPS of caged glutamate resulted in 3 responses observed in whole cell patch clamp recordings of SPNs: top traces, large amplitude direct responses with onset locked to laser pulse onset (dashed vertical blue line); middle traces, synaptic response of consistent EPSCs within the monosynaptic event window (red box); bottom traces, no consistent response with occasional spontaneous EPSCs. Scale bar for direct traces: 50pA; for synaptic and spontaneous: 25pA. (b) Local (left) and translaminar (right) glutamatergic input maps for SPNs recorded at P1-2. Pixel size: 50µm. (c) Average input profile for local (blue) and translaminar (orange) SPNs; translaminar SPNs showed increased input from the cortical plate (CP) and reduced local (L6a/SP) innervation. (d) Left panel, average input map for translaminar SPNs recorded P3-P4 (n=6); right panel, average input profile for local (blue) and translaminar (orange) SPNs. (e) corresponding input profile for P5-P6. (f) Average input map and profile for SPNs recorded between P7-P8. Horizontal profile for local and translaminar SPNs aligned on cell soma at (g) P1-P4 and (h) P5-P8. Horizontal axis indicates the lateral distance from the soma. (i) Schematic showing glutamatergic circuit onto *Lpar1-EGFP* SPNs.

**Figure 4 Cell death increases during the postnatal day (P)5-P6 window but is not confined to the subplate.**

(a) Immunohistochemistry for cleaved Caspase-3 (Casp-3) and EGFP in subplate (SP) at P4; scale bar: 25 μm. Left panel, Casp-3-positive cells in the white matter. The area bounded by the white dashed line is shown at higher magnification in the centre and right panels with Casp-3+ cells with pyknotic nuclei identified with white arrows in the centre panel; scale bar: 9 μm. (b) Similar data obtained at P6 but with white arrowhead pointing to EGFP+, Casp-3+ *Lpar1-EGFP* SPN with pyknotic nucleus. (c) Summary data for percentage *Lpar1-EGFP* SPNs exhibiting pyknotic nuclei at postnatal day (P)3-4 (n=5 animals) and P5-6 (n=5). (d) Plot showing the density of EGFP+ cells in SP at P3-4 (n=5) and P5-6 (n=5); asterisks, p=0.0327 (2 tailed t-test; t=2.579, df=8). (e,f) TUNEL staining at (e) P4 and (f) P6 with TUNEL+ cells indicated with white arrows. The area identified by the white dashed box is shown expanded in (f) in the right panels; the white arrowhead identifying a TUNEL+ *Lpar1-EGFP* SPN; scale bar:. (g) Plot of percentage TUNEL+ *Lpar1-EGFP* SPNs at P3-4 and P5-6; asterisks, p=0.0274 (2 tailed t-test; t=2.693, df=8). (h) Density of TUNEL+ cells in the white matter (WM), subplate (SP) and adjacent L5/L6a across both timepoints; single asterisk, p=0.028; double asterisk, p=0.011.

**Figure 5 GABAergic input onto Lpar1-EGFP SPNs in the first postnatal week.**

a) Top panel, synaptic and no input response observed in whole cell patch clamp recordings of SPNs voltage clamped at the approximate reversal potential for glutamate (E_Glut_). Bottom panel, recovered fusiform morphology of the SPN with translaminar GABAergic input map shown in (b). (c-e) GABAergic input onto SPNs recorded from P1-P4. Average input maps for (c) local and (d) translaminar SPNs with profile shown in (e). (f-h) corresponding data for SPNs recorded between P5-P8. (i,j) Columnar analysis of GABAergic input on SPNs at (i) P1-P4 and (j) P5-P8. (k) Schematic of GABAergic input onto fusiform and pyramidal (Pyr) SPNs present from P1-P4.

**Figure 6 Somatostatin-positive (SST+) interneurons innervate *Lpar1-EGFP* SPNs during early postnatal life**.

(a) Widefield 473nm blue light stimulation evoked IPSCs in SPNs at both P3-P4 (n=7) and P5-P6 (n=6) following conditional expression of Channelrhodopsin2 (ChR2) in SST+ interneurons. LED on indicated by the blue line. (b) Incubation in Sr^2+^-containing ACSF resulted in asynchronous neurotransmitter release. (c) Cumulative probability plot of ChR2-evoked IPSC amplitude for control (black line; n=13) versus Sr^2+^-containing HDC ACSF (grey; n=7) across P3-P6; asterisk: two-sample Kolmogorov-Smirnov test: P ≤ 0.01. (d) Comparison of early (P3-P4; n=3) versus late (P5-P6; n=4) IPSC amplitude in the presence of Sr^2+^-containing HDC ACSF. (e) Local and translaminar SST+ interneuron input maps onto SPNs revealed through LSPS uncaging of ATP in conjunction with conditional expression of P2x2 receptor in SST+ interneurons. (f) Local (blue) and translaminar (orange) average layer profiles for SST+ input onto SPNs.

**Figure 7 Sparse thalamic afferent input onto Lpar1-EGFP SPNs in postnatal S1BF.**

(a) Schematic showing the experimental set-up for recording electrical stimulation-evoked TC-EPSCs in thalamocortical slices. (b) Percentage EGFP+ SPNs exhibiting constant latency synaptic response to minimal electrical stimulation over development. (c) Box plot showing the average amplitude for EPSCs. (d) Plot of jitter (Standard deviation of onset EPSC) versus average latency for all the responses shown in (b,c). Putative TC-ESPCs had latency ≤10ms and jitter ≤1ms. (e) Plot of average EPSC amplitude (pA) versus 10-90% rise time for all putative TC-EPSCs. Dashed circle, cluster of type 1 TC-EPSCs (top, right trace) versus type 2 (bottom trace); K-means cluster analysis (median silhouette values): K=2; Sil = 0.89 +- 0.00; K=3; Sil = 0.74 +- 0.04; K=4; Sil = 0.75 +- 0.05; K=5; Sil = 0.71 +- 0.04. (f) Box plot of average jitter for type 1 and type 2 EPSCs. (g) Schematic showing the experimental set-up for combined electrical and optogenetic stimulation of thalamic afferents. (h) (top panel) Blue light reliably evoked inward currents in thalamic relay neurons following Olig3 Cre conditional expression of ChR2, sufficient to trigger action potentials in loose cell attached recordings (bottom panel). (i) Electrical (top trace) and optogenetic (bottom trace) stimulation evoked TC-EPSC in a single Lpar1-EGFP SPN at P2. Bottom panel, plot of electrical stimulation evoked TC-EPSCs recorded during the combined electrical and optogenetic stimulation experiments. Blue data points indicate the two cells that also exhibited optogenetic EPSCs; dashed blue line, TC-EPSCs with a type 1 profile.

**Figure 8 Model for Lpar1-EGFP SPN circuits in early postnatal cortex.**

(a) Pyramidal Lpar1-EGFP SPNs received broad glutamatergic (grey neurons) and GABAergic (black) input from the subplate zone. (b) Transient fusiform Lpar1-EGFP SPNs in contrast receive translaminar input from glutamatergic neurons (grey) in the cortical plate and SST+ interneurons (black) in infragranular layers. (c) We propose that sparse thalamic input (Th) onto Lpar1-EGFP SPNs interacts with both fusiform and pyramidal SPN circuits to sculpt the emergent columnar cytoarchitecture (blue dashed box).
